# UPLC-QTOF/MS Metabolomics and Biochemical Assays Reveal Changes in Hepatic Nutrition and Energy Metabolism during Sexual Maturation in Female Rainbow Trout (*Oncorhynchus mykiss*)

**DOI:** 10.3390/biology11111679

**Published:** 2022-11-18

**Authors:** Lu Ding, Yingjie Liu, Meng Kang, Xiaofeng Wei, Chuanye Geng, Wenzhi Liu, Lin Han, Fangying Yuan, Peng Wang, Bingqian Wang, Yanchun Sun

**Affiliations:** 1 Laboratory of Quality & Safety Risk Assessment for Aquatic Products (Harbin), Heilongjiang River Fisheries Research Institute of Chinese Academy of Fishery Sciences, Ministry of Agriculture and Rural Areas, Harbin 150070, China; 2 Department of Food Science and Engineering, College of Food Science and Technology, Shanghai Ocean University, Shanghai 201306, China; 3 Heilongjiang Provincial Fishery Extension Center, Harbin 150080, China; 4 Department of Food Science and Engineering, College of Food Science and Engineering, Dalian Ocean University, Dalian 116023, China; 5 Department of Chemical Engineering and Technology, College of Materials and Chemical Engineering, Harbin University of Science and Technology, Harbin 150080, China

**Keywords:** rainbow trout, sexual maturation, metabolomics, nutrition and energy metabolism

## Abstract

**Simple Summary:**

Although the close relationship between nutrition mobilization and liver metabolism has been proposed in female rainbow trout with normal sexual maturity, detailed information concerning the molecular mechanisms that the liver regulates nutrition and energy metabolism to facilitate sexual maturity remains limited. In the present study, metabolomic and biochemical assays were used to investigate differences in liver metabolic characteristics between mature and immature female rainbow trout to systematically analyze the mechanisms of hepatic nutrients and energy metabolism during sexual maturation in female rainbow trout. Our results revealed that during the sexual maturation of female rainbow trout, the liver enhanced various pathways of lipid metabolism and amino acid anabolism to provide structural substances that promote the proliferation and differentiation of oocytes. At the same time, hepatic glycogen catabolism was the primary metabolic pathway for energy production. In conclusion, this study might give a deeper insight into understanding the “nutrition-reproduction” interaction of female rainbow trout at the metabolic level.

**Abstract:**

Mobilization and repartition of nutrients and energy are prerequisites for the normal sexual maturity of broodstock. However, there are few studies on the mechanisms of hepatic nutrients and energy metabolism during sexual maturation in female rainbow trout (*Oncorhynchus mykiss*). This study investigated hepatic metabolite changes and explored the potential nutritional regulation mechanism between mature and immature female rainbow trout by combining UPLC-QTOF/MS metabolomics and biochemical assays. It was observed that hepatic biochemical assays differed considerably between the two groups, such as glucose, triglycerides, hexokinase, lipase, and aspartate aminotransferase. Liver metabolomics showed that various differential metabolites involved in amino acid and lipid metabolism markedly increased, suggesting the enhancement of lipid metabolism and amino acid anabolism in the liver provides the necessary material basis for ovarian development. Meanwhile, glycogen catabolism and glycolysis hold the key to maintaining organismal energy homeostasis with normal sexual maturation of female rainbow trout. Overall, the results from this study suggested that the liver undergoes drastic reprogramming of the metabolic profile in response to mobilization and repartition of nutrients and energy during the sexual maturation of female rainbow trout. This study further deepened the understanding of the reproductive biology of rainbow trout, and provided the theoretical basis and practical ramifications for nutritional requirements of breeding high-quality broodstock in the artificial propagation of rainbow trout.

## 1. Introduction

Rainbow trout (*Oncorhynchus mykiss*) possesses delicious meat and incredible nutritional value, which is recommended as an economical edible cold-water bony fish by the Food and Agriculture Organization of the United Nations (FAO) [1]. In the artificial propagation of rainbow trout, the production of all-female has better advantages of gender-related differences than all-male, such as growth rate, resistance to diseases, tolerance of environmental stress factors, greater homogeneity of size, and flesh quality at harvest [2]. Thus, female rainbow trout have greater economic value than males. During the gonad development of fish, there are differences in energy metabolism and nutrient utilization between females and males, and ovarian development consumes more energy and nutrient than testis. Compared with male fish, females exhibit greater lipid accumulation, lipogenesis activity and circulating glucose dependence for ovarian development and oogenesis [3,4]. At the same time, it should be mentioned that the ovaries of rainbow trout increase sharply to 20~30% of body weight, and the specific growth rate rises considerably more than that of males, as well as fillet quality in terms of lipid content and texture [5]. Consequently, female rainbow trout might have a stronger metabolic recombination capacity than males during gonadogenesis. Although rainbow trout is an important research object in aquatic genetics and breeding, it has not yet been solved in terms of artificial propagation [6], including germplasm degradation, low fecundity, and poor survival rate. On a cautionary note, the artificial hatcheries of fecundity, hatchability, and larvae survival rate heavily are closely related to the normal sexually mature females to a large extent. The majority of previous studies have shown that the energy and nutrients required for gonad development of fish are preferentially mobilized from endogenous lipid and protein stores of viscera or muscle adipose tissue [7]. It has been recognized that nutrient mobilization and repartition are the prerequisites for normal ovarian development and maturation of fish, which lay a solid foundation for the quantity and quality of offspring [8]. However, little is understood about the metabolic regulation of nutrient and energy mobilization in female rainbow trout during this period.

The liver is the central metabolic organ in response to nutritional and hormonal signals, which regulates the synthesis and metabolism of lipids, carbohydrates, and proteins [9]. During the period of sexual maturation, the liver of fish undergoes a series of dramatic physiological and biochemical changes. Semra et al. [10] found that the composition and content of fatty acids changed in the liver of *Mastacembelus mastacembelus* during sexual maturation. Bogevik et al. [11] discovered that phospholipid and LC-PUFA metabolism changed dramatically in the liver and gonad during the sexual maturation of *Salmo salar*. Cristina et al. [12] revealed changes in metabolites and energy reserves by analyzing biochemical indicators of gonad, liver and muscle during the sexual maturation of *Octopus maya*. These studies demonstrated that biochemical indicators and metabolic changes with the liver as the target organ could be used to explore the mobilization and repartition of nutrients and energy during the sexual maturation of fish. However, the current research concerning the sexual maturation of female rainbow trout predominantly focuses on the physiological histology based on ovarian appearance staging and gonadal index [13], the genetics of genes regulating gonadal differentiation [14], and the effects of certain exogenous factors [15]. Few details are known regarding how the liver coordinates changes in nutrient and energy metabolism to permit the normal sexual maturation of female rainbow trout.

Metabolomics is an emerging and powerful technology that captures changes in the metabolic spectrum and reveals the organism’s metabolic nature and microenvironmental state through a comprehensive systematic analysis [16]. It has been widely used to study the abstruse metabolic mechanisms of fish in response to changes in endogenous and exogenous factors [17]. Sun et al. [18] utilized UPLC-QTOF/MS metabolomics technology to explore the changes in biomarkers and pathways of crucian carp (*Carassius auratus*) under carbonate alkalinity exposure to reveal the molecular and physiological mechanisms of fish salt tolerance. Ladisa et al. [19] employed UHPLC-MS technology to investigate the seasonal metabolic changes and energy distribution associated with the growth and reproductive stages of the liver of male goldfish (*Carassius auratus*). Yi et al. [20] applied a combination of UPLC-MS/MS metabolomics to investigate the metabolites and genes related to ovarian maturation and spawning in blunt snout bream (*Megalobrama amblycephala*). Ultra-performance liquid chromatography coupled with quadrupole time-of-flight mass spectrometry (UPLC-QTOF/MS) has become the mainstream analytical technology of metabolomics in terms of its high sensitivity and resolution [21]. However, there is little previous research utilizing UPLC-QTOF/MS metabolomics technology to explore the liver metabolic mechanisms responsible for the mobilization and repartition of nutrients and energy during the sexual maturation of female rainbow trout.

In the present study, UPLC-QTOF/MS metabolomics coupled with biochemical indicators were performed to systematically analyze the differences in the composition and content of hepatic metabolites between mature and immature female rainbow trout. Furthermore, we elucidated how the mobilization and repartition mechanisms of nutrients and energy in the liver of female rainbow trout during sexual maturation. This study would give a deeper insight into the “nutrition-reproduction” interaction of female rainbow trout, and provide a theoretical basis and practical ramifications for nutritional requirements and precise feeding of rainbow trout broodstock in artificial propagation.

## 2. Materials and Methods

### 2.1. Materials

HPLC-grade methanol, acetonitrile, and formic acid were acquired from Merck (Darmstadt, Germany) and Anpel (Shanghai, China). Tricaine Methanesulfonate (MS-222) was purchased from Sigma Aldrich (St. Louis, MO, USA). The kits of glucose (Glu), triglycerides (TG), total cholesterol (TC), hexokinase (HK), lipase (LPS), total protein (TP), aspartate aminotransferase (AST) and alanine aminotransferase (ALT) were obtained from Nanjing Jiancheng Institute of Biological Engineering (Nanjing, China).

### 2.2. Experimental Fish and Sampling

The rainbow trout were provided by the Bohai Experiment Station of Heilongjiang Fisheries Research Institute, Chinese Academy of Fishery Sciences. Randomly selected healthy 2-year-old rainbow trout (365.70.8 ± 36.89 g, 27.53 ± 1.32 cm) and 3-year-old rainbow trout (1054.00 ± 49.67 g, 37.79 ± 1.68 cm) for the experiment. After being anesthetized with MS-222 (40 mg/L), the fish were immediately dissected on ice. Furthermore, the gonad development stages of this experimental fish were identified according to Meng’s method [22]. Finally, the gonad development stage of 2-year-old female rainbow trout was categorized as stage II (immature stage), which belonged to immature individuals (named as IMR group), and that of 3-year-old females was stage IV and V (mature and spawning stage), which belonged to mature individuals (named as MR group). Twenty-five female rainbow trout were randomly selected from the IMR and MR groups, respectively, and their liver tissue was immediately collected, packaged and frozen in liquid nitrogen, and stored at −80 °C for further use. In subsequent analyses, 18 livers were used for UPLC-QTOF/MS metabolomics and 7 for biochemical analysis.

### 2.3. Biochemical Parameters’ Determination

The liver tissue samples with ice-cold physiological saline (1:9, *w*/*v*) were homogenized at −20 °C, 60 Hz. The obtained homogenate was centrifuged at 2500× *g*, 4 °C for 10 min, and the supernatant was collected. According to the kit manufacturer’s instructions, glucose (Glu), triglycerides (TG), total cholesterol (TC), hexokinase (HK), lipase (LPS), total protein (TP), aspartate aminotransferase (AST) and alanine aminotransferase (ALT) were measured.

### 2.4. Metabolomics Analysis

#### 2.4.1. Sample Preparation

We weighed 90 mg of the thawed liver tissue sample accurately and added 1200 uL of pre-chilled organic extraction solvent (methanol: water = 4:1, *v*/*v*) for extraction. It was then homogenized at −20 °C, 60 Hz for 2 min, ultrasonicated at 4 °C for 30 min, set at −20 °C for 30 min, and centrifuged at 4 °C, 13,000 rpm for 15 min. An amount of 600 uL of the supernatant was taken and filtered into a liquid chromatography glass vial through a 0.22 μm organic-phase filter membrane for UPLC-QTOF/MS analysis. In addition, a 50 μL amount of each sample was mixed into the quality control (QC) sample, which was applied to monitor the deviation of the analysis result from the mixed sample and the error caused by the instrument itself.

#### 2.4.2. UPLC-QTOF/MS Conditions

The chromatographic separation was performed on the pre-column of Acquity UPLC BEH C18 (2.1 mm × 100 mm, 1.7 μm) and AQUITY UPLC BEH C18 (2.1 mm × 5 mm, 1.7 μm) (Waters, Milford, MA, USA). The mobile phase A was 0.1% formic acid in water (*v*/*v*), and the mobile phase B was 0.1% formic acid in acetonitrile (*v*/*v*). The gradient elution procedure was performed as follows: 0~2 min, 5~20% B; 2~3 min, 20~60% B; 3~11 min, 60~80% B; 11~12 min, 80~100% B; 12~13 min, 100~100% B; 13~13.1 min, 100~5% B; 13.1~15 min, 5~5% B. The column and autosampler tray temperatures were maintained at 30 °C and 4 °C, respectively. The flow rate was set to 0.3 mL/min, and the injection volume was set to 10 μL.

Mass spectrometry analysis used a Triple TOF 5600^+^ system (SCIEX, Framingham, MA, USA) with an electrospray ionization (ESI) source. The TOF/MS conditions were as follows: ion spray voltage was set to 5000 V (ESI+) or 4500 V (ESI−), ion source temperature was 550 °C, declustering potential was ±80 V, collision energy was ±35 eV, and collision energy spread was ±15 eV. Nitrogen was used as an atomizing gas and auxiliary gas. The pressure of Gas1 and Gas2 was 55 psi, and the curtain gas was 35 psi. The TOF-MS scan was configured to acquire the MS fragment ions, and the mass scan range was 100–1200 Da. The information-dependent acquisition (IDA) was configured to acquire the MS/MS fragment ions and the mass scan range was 50–1200 Da.

### 2.5. Data Processing and Statistical Analysis

GraphPad Prism 9.0 (GraphPad, San Diego, CA, USA) and OriginPro 2021 (OriginLab, Northampton, MA, USA.) were applied to analyze the biochemical assay data. The Student’s *t*-test was performed to detect significant differences between the two groups, and the results were denoted as the mean ± SD, N = 7, asterisks (*) indicate statistically significant differences among the groups (* *p* < 0.05, ** *p* < 0.01 and *** *p* < 0.001).

The Progenesis QI software (Waters, Milford, MA, USA) was used to complete the preprocessing of the collected raw data of UPLC-QTOF/MS, including peak alignment, peak picking, peak identification, and normalization. We used the following parameters: precursor tolerance was set to 5 ppm, fragment tolerance was set to 10 ppm, and retention time (RT) tolerance was set to 0.02 min. The noise elimination level was set at 10.00, and the minimum intensity was set to 15% of the base peak intensity. Metabolites were annotated using HMDB and LIPIDMAPS public databases (http://www.hmdb.ca/ accessed on 18 April 2022), http://www.lipidmaps.org/ accessed on 18 April 2022). Finally, a three-dimension data set including m/z, RT, peak intensity, sample name, and metabolite name as output, and RT-m/z pairs were used as the identifier for each ion. The data matrix was imported into SIMCA 14.1 software (Umetrics, Umea, Sweden) for pattern recognition, including principal component analysis (PCA) and orthogonal partial least squares discriminant analysis (OPLS-DA) to determine the overall metabolic changes between the IMR group and the MR group. The metabolites were identified as differential metabolites (DMs) under the conditions of variable importance in projection (VIP) > 1 and *p* < 0.05. MetaboAnalyst 5.0 (http://www.metaboanalyst.ca/ accessed on 18 April 2022) was used to determine the biological pathways related to DMs, integrating enrichment analysis and pathway topology analysis [23].

## 3. Results

### 3.1. Biochemical Indicators

**Figure 1:**
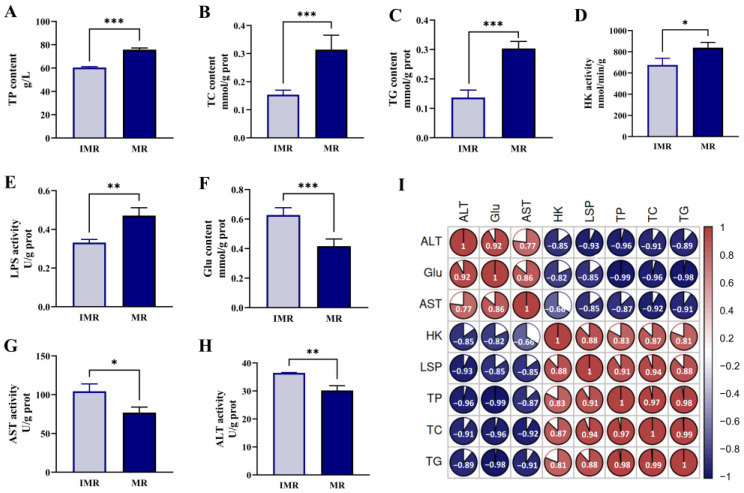
Changes in hepatic biochemical indicators between immature female rainbow trout and mature females. (**A**) total protein (TP), (**B**) total cholesterol (TC), (**C**) triglycerides (TG), (**D**) hexokinase (HK), (**E**) lipase (LPS), (**F**) glucose (Glu), (**G**) aspartate aminotransferase (AST), (**H**) alanine aminotransferase (ALT). IMR represents immature female rainbow trout; MR represents mature female rainbow trout. The data are presented as the means ± SD, and asterisks indicate statistically significant differences among the groups (* *p* < 0.05; ** *p* < 0.01; *** *p* < 0.001). (**I**) Pearson correlation analysis between the biochemical indicators, the size of the pie chart represents the magnitude of the correlation, red and blue represent positive and negative correlations, respectively.

As a result of the study, we determined that the hepatic biochemical indexes differed remarkably between immature and mature female rainbow trout. Compared with the IMR group, there were statistically significantly higher levels of TP, TC, TG, HK and LPS (Figure 1A–E), and conversely, the contents of Glu and the activities of AST and ALT tended to be decreased in the MR group (Figure 1F–H). Pearson correlation analyses were performed to assess the correlation between pairs of hepatic biochemical parameters (*p* < 0.05). As shown in Figure 1I, there was a significant inverse correlation between Glu and lipids, and HK was positively correlated to LPS, indicating the liver would consume a lot of glucose and enhance lipid metabolism providing energy and structural support for the normal development of ovaries during the sexual maturation of female rainbow trout.

### 3.2. Metabolic Profiles

**Figure 2:**
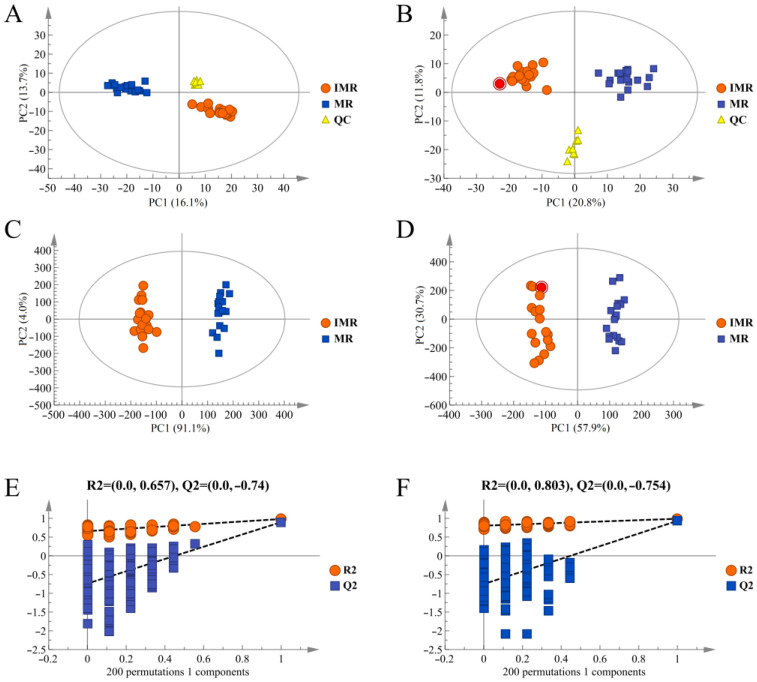
Changes in the hepatic metabolic profile between immature female rainbow trout and mature females. (**A**) The PCA scores plot in the positive ion mode. (**B**) The PCA scores plot in the negative ion mode. (**C**) The OPLS-DA score plot in the positive ion mode. (**D**) The OPLS-DA score plot in the negative ion mode. (**E**) The OPLS-DA permutation test in the positive ion mode. (**F**) The OPLS-DA permutation test in the negative ion mode. IMR represents immature female rainbow trout; MR represents mature female rainbow trout.

The liver metabolic profiles were obtained by UPLC-QTOF/MS in positive and negative-ion modes, and the differences between the IMR and MR samples among groups were detected by PCA and OPLS-DA. The PCA scores between QC, IMR and MR samples showed a clear separation and the same biological sample located robust clustering, indicating that there were considerable differences in the metabolites between mature and immature female rainbow trout, and the instrument had high stability and reliability (Figure 2A,B). The supervised OPLS-DA model was carried out to further determine differential metabolites during the sexual maturation of the female rainbow trout. The OPLS-DA model quality parameters were: R^2^(cum) = 0.976, Q^2^(cum) = 0.926 in positive-ion mode; R^2^(cum) = 0.981, Q^2^(cum) = 0.896 in negative-ion mode. R^2^ and Q^2^ were greater than 0.5, demonstrating that the OPLS-DA model had a high accuracy of the representative model fit and interpretation, and could be exploited in the subsequent analyses (Figure 2C,D). In the permutation test, the OPLS-DA model’s original values of R^2^ and Q^2^ were larger than those generated from the random permutations, verifying that the established OPLS-DA model was not overfitted and statistically reliable (Figure 2E,F).

### 3.3. Identification of Differential Metabolites

**Figure 3:**
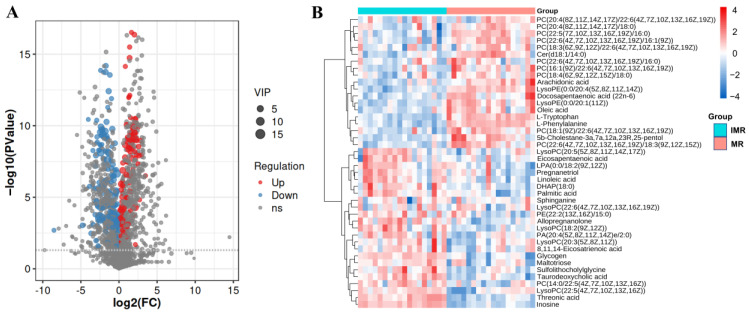
The hepatic differential metabolites change between immature female rainbow trout and mature females. (**A**) Volcano map of the DMs. (**B**) Heat map visualization of the DMs.

We used the fold-change cutoff of 2, VIP > 1, and *p* < 0.05 to identify the distribution of metabolites between the IMR group and MR group. As shown in Figure 3A, the volcano plots show the differential abundance of metabolites before and after the sexual maturity of female rainbow trout. A total of 42 DMs (VIP > 1 and *p* < 0.05) were identified and divided into 21 glycerophospholipids, 8 fatty acids, 4 steroids and steroid derivatives, 4 carbohydrates, 2 amino acids, 2 sphingolipids and 1 nucleoside (Table S2). Compared with the IMR group, 24 DMs were up-regulated, and 18 DMs were down-regulated in the MR group. Hierarchical clustering analysis showed a significant color distinction between the IMR group and MR group, indicating that these DM contents varied considerably before and after sexual maturity in female rainbow trout (Figure 3B).

### 3.4. Metabolic Pathway Analysis

**Figure 4:**
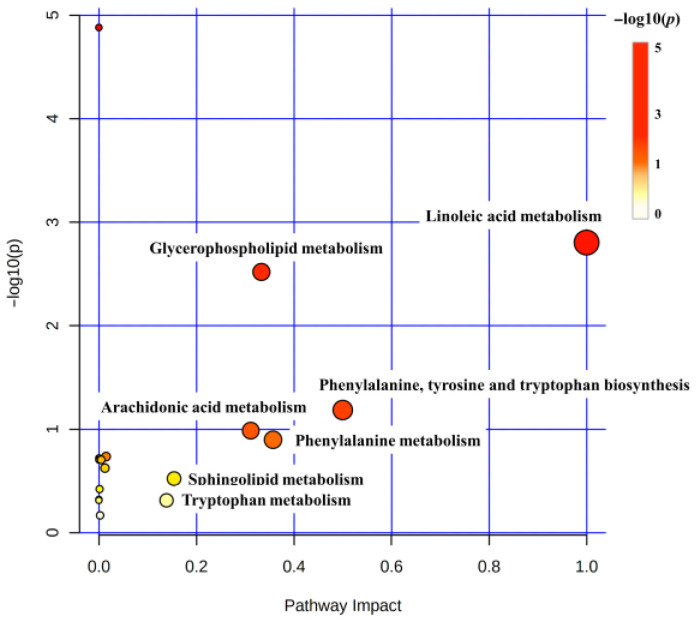
The hepatic pathway analysis based on MetaboAnalyst 5.0. Bubble size is proportional to the impact of each pathway, and bubble color denotes the degree of significance from the highest (red) to the lowest (white).

**Figure 5:**
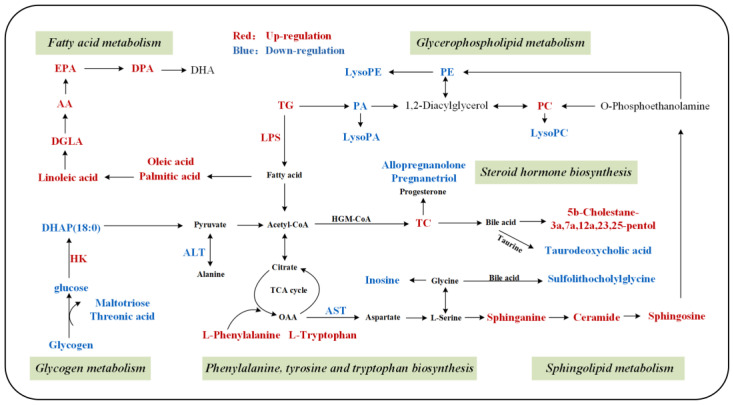
The hepatic metabolic pathway network based on the KEGG database during the sexual maturation of female rainbow trout. Red and blue represent hepatic metabolites that statistically significantly increased and decreased, respectively.

DMs were introduced into MetaboAnalyst 5.0 to discern the potentially biologically meaningful metabolic pathways. The DMs were annotated into 17 metabolic pathways. Based on −log10(*p*) and pathway impact scores, we further identified seven key metabolic pathways, including linoleic acid metabolism, glycerophospholipid metabolism, phenylalanine, tyrosine and tryptophan biosynthesis, arachidonic acid metabolism, phenylalanine metabolism, sphingolipid metabolism, and tryptophan metabolism (Figure 4). Ultimately, integrating the correlations of DMs with KEGG analysis, we summarized and manually drew a metabolite–pathway interaction network in the liver, which was considered potentially associated with the mobilization and repartition of nutrients and energy during the sexual maturity of female rainbow trout (Figure 5).

## 4. Discussion

Rainbow trout is one of the economically valuable aquaculture commodities around the world. Female fish with normal maturation have been shown to greatly improve offspring of artificial propagation quality and production [20]. It is agreed upon that female sexual maturity is susceptible to energy metabolism, which relies on the mobilization of stored carbohydrates, lipids and proteins [24]. The liver responds to hormones and metabolic signals as the center of energy metabolism, which is indispensable for sexual maturation in fish [9]. Consequently, the fish liver undergoes a series of dramatic physiological and metabolic changes during maturation. However, hepatic energy metabolism and nutrient distribution characteristics remain ill-defined during sexual maturation in female rainbow trout. In this study, UPLC-QTOF/MS metabolomics combined with biochemical parameters revealed that the liver responds to nutrient and energy mobilization and redistribution by regulating lipid metabolism, carbohydrate metabolism, and amino acid metabolism during the sexual maturation of female rainbow trout.

### 4.1. Lipid Metabolism

Lipids participate in various physiological and biochemical processes, including triglycerides, cholesterol, glycerophospholipids, sphingolipids and fatty acids, whose storage and utilization are critical to maintaining cellular energy homeostasis [25]. Lipid metabolism provides organisms with the necessary energy and precursor substances and ensures the orderly progress of various physiological, developmental, and reproductive processes. In this study, the identified lipid DMs were classified into fatty acids, glycerophospholipids, sphingolipids, steroids and steroid derivatives, which were involved in linoleic acid metabolism, arachidonic acid metabolism, glycerophospholipid metabolism, sphingolipid metabolism, and steroid hormone biosynthesis.

#### 4.1.1. Fatty Acid Metabolism

Triglyceride (TG), the important form of fish energy storage, can be hydrolyzed into glycerol and fatty acids (FAs) under a series of lipases (LPS) to participate in material and energy metabolism [26]. In this study, fatty acids including palmitic acid, oleic acid, linoleic acid (LA), arachidonic acid (AA), 8,11,14-eicosatrienoic acid (DGLA), eicosapentaenoic acid (EPA), and docosapentaenoic acid (DPA) were significantly different in the liver of mature and immature female rainbow trout. These substances are mainly involved in unsaturated fatty acid biosynthesis, linoleic acid metabolism, and arachidonic acid metabolism. The contents of TG and FAs and the activities of LPS were significantly increased in the liver of mature females compared to immature female rainbow trout. Previous studies have shown that increased FAs in the liver of female fish not only serve as an energy source, but also as substrates for vitellogenin synthesis and deposition in the developing oocytes [5]. Consequently, during the sexual maturation of female rainbow trout, the liver of female rainbow trout has a greater lipid accumulation and lipid catabolism activity to produce a high level of fatty acids and energy for promoting both oocyte nuclear and cytoplasmic maturation [27].

Polyunsaturated fatty acids are not only the preferred substrates during hepatic β-oxidation, but also intermediates in the biosynthesis of hormones and other signaling molecules [20]. In fish, LA is first metabolized by Δ6-desaturase into γ-linolenic acid and DGLA, and then forms the crucial intermediate metabolite AA, which can be catalyzed by cyclooxygenase to EPA or eicosanoids [28]. As a precursor of eicosanoids such as prostaglandins, AA has a variety of functions in fish reproduction, which covers pheromone expression, ovulation induction, and steroid production [29]. DPA is a metabolic intermediate of EPA and DHA [30]. EPA is further metabolized into DHA or eicosanoids promoting oocyte maturation and ovulation [31]. DHA plays an important role in maintaining fish’s vision, nervous system, and reproduction [32]. A previous study on devil stinger (*Inimicus japonicas*) reported that the DHA content of the gonads was remarkably higher than other tissues during sexual maturation [33]. In this study, although there was no significant difference in DHA found in the liver of mature and immature female rainbow trout, EPA and DPA in the liver of mature individuals were significantly higher than those of immature individuals, which belonged to the precursor of DHA. Notably, Zhu et al. [32] injected stable isotope-labeled DHA into female zebrafish to facilitate tracking, which found that DHA would be transported from the liver or muscle to the ovaries during maturation. Therefore, DHA is the appropriate nutrient for developing oocytes, and the ovary may have a physiological priority for mobilizing DHA from the liver during the ovarian development of rainbow trout.

#### 4.1.2. Phospholipid Metabolism

Glycerophospholipids and sphingolipids are indispensable components for generating cell membranes, which have emerged as crucial players participating in material transport, energy conversion, and information transmission [34]. In the process of glycerophospholipid metabolism, phosphatidylcholines (PCs), phosphatidylethanolamines (PEs), and phosphatidic acids (PAs) can be catalyzed by phospholipase to generate fatty acids (FAs) and lysophospholipids, which not only provide energy to maintain normal cell growth, but also participate in the biosynthesis of DNA, RNA and eicosanoids, and promote the rapid growth and reproduction of the body [35]. Ceramide (Cer) is the central hub of sphingolipid metabolism, which can synthesize sphingolipids under the catalysis of sphingomyelin synthase, and can also be hydrolyzed by ceramidase to generate sphingosine (Sph) and FAs [36]. Cer and Sph have been documented to have various established regulatory effects on the biosynthesis of steroid hormones [37]. The present study found that compared with immature female rainbow trout, PCs were up-regulated as a whole and participated in various metabolic pathways such as glycerophospholipid metabolism, linoleic acid metabolism, alpha-linolenic acid metabolism, and arachidonic acid metabolism. Cer and Sph were up-regulated in the sphingolipid metabolism pathway. Those results were consistent with previous studies on blunt snout bream [20], Japanese catfish (*Silurus asotus*) [38] and goldfish (*Carassius auratus*) [39]. Therefore, female rainbow trout will accelerate the liver metabolism of glycerophospholipids and sphingolipids during sexual maturation. Numerous synthetic PCs could provide the structural basis for germ cell proliferation and differentiation, ceramide and sphingosine metabolites could promote the synthesis of steroid hormones, which are beneficial to normal ovarian development.

#### 4.1.3. Steroid Hormone Biosynthesis

As the basic component of biological membranes, cholesterol not only plays an important role in membrane transport, material transport and signal transduction, but also is an essential precursor of steroid hormones, vitamin D3 and bile acids [40]. The liver is a momentous organ involved in de novo cholesterol synthesis and catabolism [41]. The conversion of cholesterol to pregnenolone is the first committed step in steroid synthesis through the actions of the hepatic mitochondrial cytochrome P450 side chain lyase [42]. Progesterone (PG) is the predominant pregnancy hormone in females during the estrus cycle and pregnancy [43]. Previous studies have detected that the level of cholesterol and PG increase significantly in the blood during fish’s maturation and reproductive cycle [20,44,45]. In this study, cholesterol levels in the liver of mature female rainbow trout were significantly higher than those of immature females, thereby accelerating steroid hormone biosynthesis and oocyte development in mature female rainbow trout. However, it should be noted that, unlike previous blood studies, the levels of alloprogesterone and pregnatriol, the metabolites of PG, in female rainbow trout were significantly lower in this study than in immature female rainbow trout (*p* < 0.05). We speculated that this might be due to the fact that PG synthesized by the liver was transferred to peripheral tissues such as the blood and ovaries to maintain steroid hormone homeostasis or converted to certain substances guaranteeing oocyte development [46].

### 4.2. Carbohydrate Metabolism

Carbohydrates not only provide energy for fish movement, growth, reproduction and thermoregulation, but participate in the biosynthesis of nucleic acids, ovarian pigments and amino acids [47]. During the growth and development of fish, carbohydrates are consumed in the form of glucose transported through the blood to the corresponding site for oxidative energy production, and the excess part will be stored in the liver and muscle tissue in the form of glycogen [48]. Enzyme activities in carbohydrate metabolism hold the key to energy mobilization when the nutrient supply is deficient [49]. Hexokinase (HK) catalyzes the first step of glycolysis, whose activity serves a crucial function in maintaining organismal energy homeostasis [50]. Soengas et al. [51] reported that hepatic glycolytic activity considerably increased and hepatic glycogen contents decreased at early vitellogenesis, and the ovary had an increased use of exogenous glucose and increased gluconeogenesis during vitellogenesis of female turbot (*Scophthalmus maximus*), which might be related to protein and lipid synthesis of germ cells. In the current study, compared with immature rainbow trout, the activity of HK increased and the glucose content was remarkably decreased in the liver of mature females. Moreover, the contents of metabolites related to carbohydrate metabolism were largely down-regulated, including glycogen, maltotriose, and the intermediate product DHAP (18:0) of the glycolytic pathway. Notably, a recent study on female mantis shrimp Oratosquilla oratoria (*Stomatopoda*) also showed that glycogen was transferred from the hepatopancreas and muscle to the ovary to ensure an adequate energy supply for germ cells, which was consistent with our study result [52]. Based on the above results, we concluded that the liver might undergo carbohydrate catabolism, and provide a large amount of energy for germ cell development through glycogen catabolism and glycolysis during the sexual maturation of female rainbow trout.

### 4.3. Amino Acid Metabolism

As signal molecules with various biological functions, amino acids play fundamental roles in protein synthesis, energy metabolism, and hormone metabolism [53]. Phenylalanine is not only converted into neurotransmitter precursor tyrosine, but also involved in the TCA cycle, carbohydrate metabolism and lipid metabolism to promote fish growth and development [54]. Tryptophan is the main source of serotonin, which regulates metabolic networks such as protein synthesis, oxidative stress, immune response, and inflammation, and is also a key nutrient for gonadal development in fish [55]. Previous studies have shown that serum tyrosine, dopamine, and intermediates of tryptophan contents reach the highest in stage III of ovarian development in Chinese sturgeon reproduction [56]. In addition, a recent study on sea cucumber (*Apostichopus japonicus*) showed that the abundance of metabolites involved in phenylalanine metabolism before spawning was significantly higher than that after spawning, and phenylalanine metabolism could significantly regulate the reproductive process [57]. In this study, phenylalanine and tryptophan were significantly up-regulated and the content changes were three times in the liver of mature female rainbow trout compared to immature females, indicating that the enhancement of the phenylalanine and tryptophan metabolism could promote the biosynthesis of neurotransmitters and hormones, which is beneficial to ovarian development in female rainbow trout.

Alanine aminotransferase (ALT) and aspartate aminotransferase (AST) are involved in the decomposition and synthesis of amino acids and play an important role in protein metabolism [50]. Previous studies have shown that during the spawning period of brown trout (*Salmo trutta*), the protein concentration in serum increased and AST activity decreased [58]. In addition, Akhavan et al. [59] also found that total protein levels increased in the plasma of Sterlet sturgeon (*Acipenser ruthenus*) during ovarian development. In our study, compared with immature female rainbow trout, the activities of AST and ALT in the liver of mature female rainbow trout were significantly decreased, and the TP content was significantly increased. The results suggested that the liver structure changed, and the reduction in amino acid catabolism was conducive to protein synthesis, which was necessary for energy substrate and structural components during the sexual maturation of female rainbow trout.

## 5. Conclusions

In summary, our study showed that changes in hepatic metabolism and biochemical content were closely related to ovarian development during the sexual maturation of female rainbow trout. High levels of hepatic lipid catabolism, glycogen catabolism, and protein anabolism provide energy and structural substances for germ cell proliferation and differentiation. Notably, DHA, alloprogesterone and pregnatriol content decreased significantly in mature female rainbow trout, and the ovary might have a physiological priority to utilize them from the liver for the ovarian development of rainbow trout. This research would increase our understanding of the reproductive biology of rainbow trout and provide valuable information for further research. In addition, some verification experiments are necessary to determine the role of these differential metabolites in future research, which would further reveal the metabolic regulation mechanism of female rainbow trout liver on ovarian development.

## Data Availability

All data generated by this study are available in this manuscript and the accompanying supplementary materials.

## Supplementary Materials

The following supporting information can be downloaded at: https://www.mdpi.com/article/10.3390/biology11111679/s1, Figure S1: Total ion chromatograms (TIC) of the liver samples; Figure S2: The OPLS-DA S-plot of the liver samples; Table S1: The data matrix for PCA and OPLS-DA analysis; Table S2: The identified significantly different metabolites between immature female rainbow trout and mature females.
