# Supplementary material for: UPLC-QTOF/MS Metabolomics and Biochemical Assays Reveal Changes in Hepatic Nutrition and Energy Metabolism during Sexual Maturation in Female Rainbow Trout (Oncorhynchus mykiss)
Source: Biology (Basel). 2022 Nov 18;11(11):1679. doi: 10.3390/biology11111679 (PMC9687450; doi:10.3390/biology11111679)
Supplement: Supplementary file 1 [file biology-11-01679-s001.zip › Figures S1 and S2.pdf]

Supplementary Materials for

UPLC-QTOF/MS metabolomics and biochemical assays reveal changes in hepatic nutrition and energy metabolism during sexual maturation in female rainbow trout (*Oncorhynchus Mykiss*)

Lu Ding [1,2,†], Yingjie Liu [1,2,†], Meng Kang [5], Xiaofeng Wei[1,3], Chuanye Geng[1,2], Wenzhi Liu [1,2], Lin Han[1,2], Fangying Yuan[1,4], Peng Wang[1], Bingqian Wang[1], Yanchun Sun [1,2,3,4]

[1] *Heilongjiang River Fisheries Research Institute of Chinese Academy of Fishery Sciences, Laboratory of Quality & Safety Risk Assessment for Aquatic Products (Harbin), Ministry of Agriculture and Rural Areas, Harbin, 150070, China.*

[2] *College of Food Science and Technology, Shanghai Ocean University, Shanghai, 201306, China.*

[3] *College of Food Science and Engineering, Dalian Ocean University, Dalian, 116023, China.*

[4] *College of Materials and Chemical Engineering, Harbin University of Science and Technology, Harbin 150080, China.*

[5] *Heilongjiang Provincial Fishery Extension Center, Harbin 150080, China*

[*] Correspondence: sunyc2004@163.com (Y.C. Sun).

[†] These authors contributed equally to this work

*Corresponding authors at: Heilongjiang River Fisheries Research Institute of Chinese Academy of Fishery Sciences, Laboratory of Quality & Safety Risk Assessment for Aquatic Products (Harbin), Ministry of Agriculture and Rural Areas, Harbin, 150070, China.

E-mail addresses: sunyc2004@163.com (Y.C. Sun).

Reprint requests to Tel: +86-0451-84861316. Fax: +86 0451 84604803.

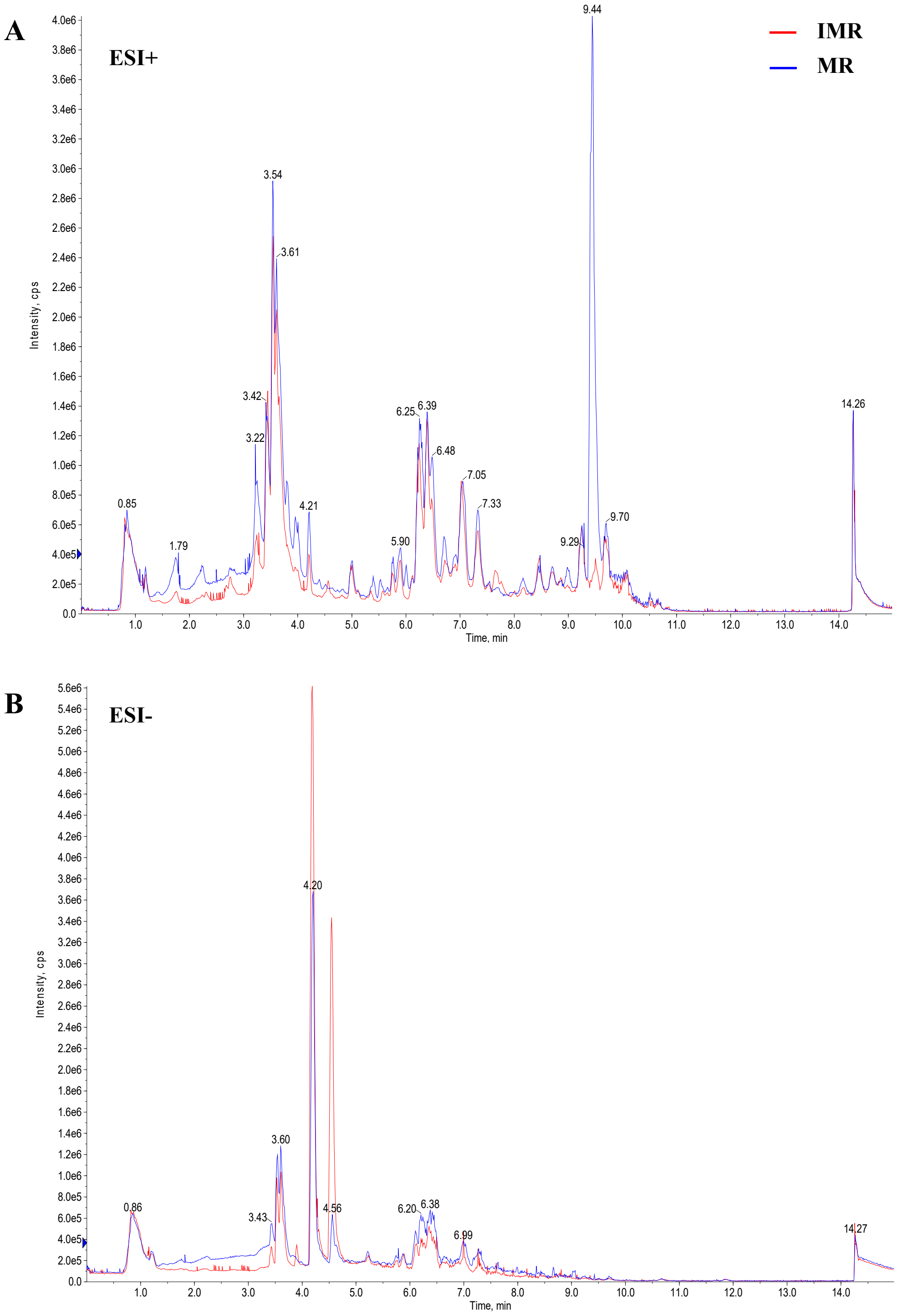

Figure S1. Total ion chromatograms (TIC) of live samples in the positive ion (A) and negative ion (B) models.

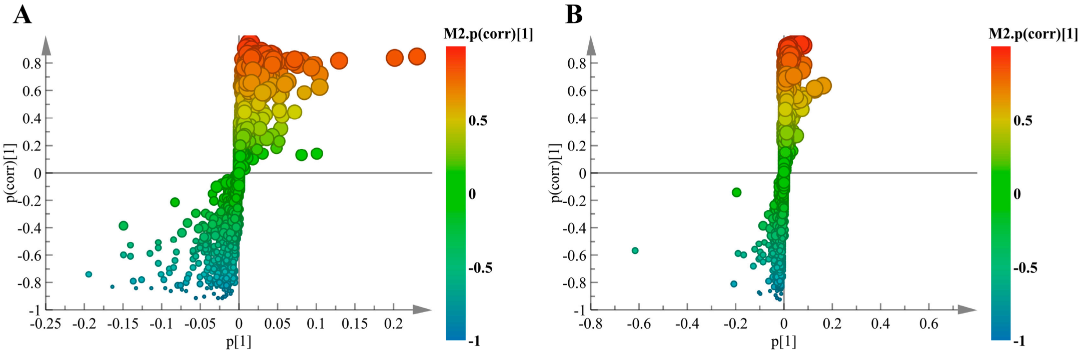

Figure S2. The OPLS-DA S-plot of positive ion (A) and negative ion (B) models.
